# Collective self-understanding: A linguistic style analysis of naturally occurring text data

**DOI:** 10.3758/s13428-022-02027-8

**Published:** 2022-11-28

**Authors:** Alicia Cork, Richard Everson, Elahe Naserian, Mark Levine, Miriam Koschate-Reis

**Affiliations:** 1 Department of Psychology, University of Bath, Bath, UK; 2 Department of Computer Science, University of Exeter, Exeter, UK; 3 Institute for Data Science and Artificial Intelligence, University of Exeter, Exeter, UK; 4 Alan Turing Institute, London, UK; 5 Department of Politics, University of Exeter, Exeter, UK; 6 Department of Psychology, University of Lancaster, Lancaster, UK; 7 Department of Psychology, University of Exeter, Exeter, UK

**Keywords:** Group identities, Linguistic style analysis, Multidimensional scaling, Naturally occurring text data

## Abstract

Understanding what groups stand for is integral to a diverse array of social processes, ranging from understanding political conflicts to organisational behaviour to promoting public health behaviours. Traditionally, researchers rely on self-report methods such as interviews and surveys to assess groups’ collective self-understandings. Here, we demonstrate the value of using naturally occurring online textual data to map the similarities and differences between real-world groups’ collective self-understandings. We use machine learning algorithms to assess similarities between 15 diverse online groups’ linguistic style, and then use multidimensional scaling to map the groups in two-dimensonal space (*N*=1,779,098 Reddit comments). We then use agglomerative and k-means clustering techniques to assess how the 15 groups cluster, finding there are four behaviourally distinct group types – vocational, collective action (comprising political and ethnic/religious identities), relational and stigmatised groups, with stigmatised groups having a less distinctive behavioural profile than the other group types. Study 2 is a secondary data analysis where we find strong relationships between the coordinates of each group in multidimensional space and the groups’ values. In Study 3, we demonstrate how this approach can be used to track the development of groups’ collective self-understandings over time. Using transgender Reddit data (*N*= 1,095,620 comments) as a proof-of-concept, we track the gradual politicisation of the transgender group over the past decade. The automaticity of this methodology renders it advantageous for monitoring multiple online groups simultaneously. This approach has implications for both governmental agencies and social researchers more generally. Future research avenues and applications are discussed.

## Introduction

Much of our social existence is impacted by the groups to which we belong, and the in- and outgroup distinctions that we make. Understanding what groups stand for is therefore of great importance to a wide range of scholars in the social sciences. For instance, on the societal level, it allows political scientists, sociologists and psychologists better insight into social phenomena such as emerging political movements (e.g., Bednarek-Gilland, 2015; Van Bavel & Packer, 2021), shifts in power structures, national and international conflicts (McCann et al., 2020; Smith, 2021; Thome, 2015), the impact of groups on sustainability (Horcea-Milcu et al., 2019; Udall et al., 2020) and public health behaviours (Cruwys et al., 2020; Wakefield et al., 2019), radicalisation (Hogg, 2021; Smith et al., 2020), and group processes such as cohesion and social belonging (Buhrmester et al., 2018; Healy, 2019). On an organisational level, the alignment between employees’ values and the values held by the company or specific work teams has been shown to affect positive working outcomes including productivity and work satisfaction (Chung, 2017; van Knippenberg & Hogg, 2018). However, social scientists currently have to rely on relatively small-scale survey and interview data that often focus on a single group outside the wider social context, to assess a group’s collective self-understanding. Here, we propose a computational method that uses naturally occurring text data from a range of groups to understand similarities and differences in collective self-understanding.

Collective self-understanding reflects the basic norms and abstract values that a group holds, along with the groups’ purpose (e.g., whether the group is a collective action group or organisational group). The collective self-understanding is therefore an abstract understanding of who the group is and what the group stands for. Importantly, it does not comprise the specific attitudes or opinions that the group holds – it can instead be thought of as the general ‘personality’ or ‘essence’ that drives the groups’ behaviours.

For the most part, a group’s collective self-understanding is assessed through qualitative interviews or quantitative surveys (e.g., Calderon et al., 2000; Schwartz, 2012). These methods rely on self-reports of opinions, values, and goals by group members. However, introspection is notoriously challenging due to both the abstract nature of norms and values as well as the explicit questions being asked (Boyd et al., 2015). For example, Boyd and colleagues detail the difficulties of assessing values through quantitative surveys that aim to reduce abstract notions such as the value to ‘work hard’ or ‘be a good mother’ into pre-defined categories (e.g., the ten Schwartz values; Schwartz, 1992).

In this paper, we propose a methodological approach that uses linguistic style analysis to examine the direct enactment of a group’s collective self-understanding in naturally occurring text data. Linguistic style refers to how a message is communicated rather than what specifically the content of a message is. Style therefore relates to the types of words that may be used to communicate a message, such as pronouns, adverbs, words with more than six letters or filler words (the full list of word categories used to measure linguistic style can be found in Appendix 1). We argue that the way in which group members communicate with each other reveals significant cues as to the norms and values that the group holds and the type of group it is. Further, we suggest that by using linguistic data, we are able to circumvent issues associated with introspection and pre-defined categories. Instead, this methodology takes a much more abstract approach by disregarding the explicit content of communication (i.e., the specific aims, opinions and policies that are expressed) and instead focuses on the way in which ideas are communicated. By concentrating on the *style* of communication rather than the content, we argue that we are able to access more subtle cues as to the psychological reality of group members (Pennebaker, 2011). Moreover, in this paper we demonstrate that this approach allows us to compare the similarities and differences between a wide range of overlapping groups such as vocational, political or relational groups. By using naturally occurring linguistic data to study collective self-understanding, we also demonstrate how this approach can enable us to track potential changes or developments in collective self-understanding over extended periods of time.

### Linguistic style research

Whilst the majority of linguistic research has focused on the semantics of communication, a more recent trend has shifted towards the analysis of linguistic style due to its ubiquity in all communication (Boyd & Pennebaker, 2017; Pennebaker, 2011; Pennebaker et al., 2003; Tausczik & Pennebaker, 2010). In lay terms, style refers to ‘how’ a message is communicated, as opposed to ‘what’ is being said. A message can be articulated in many different ways whilst still retaining its meaning, and thus the stylistic (non-semantic) words used to convey a particular message are thought to be integral to understanding how individuals construct their own realities (Chung & Pennebaker, 2007; Pennebaker, 2011). Based on this assertion, a plethora of research has identified the link between the way a person communicates and their individual personality (Boyd & Pennebaker, 2017; Mairesse et al., 2007; Tong et al., 2020), individual values (Boyd et al., 2015) and psychopathologies (Junghaenel et al., 2008). In addition to this, researchers have also studied the link between style and demographic factors such as gender (Newman et al., 2008) and age (Löckenhoff et al., 2008).

However, more recent research suggests that linguistic style can be used to understand individuals’ changing social realities and social group memberships. In both Cork et al. (2020) and Koschate et al. (2021), the authors find that linguistic style analysis can be used to understand group processes and identities. For example, Koschate et al. (2021) find that an individual that communicates as a parent uses a different linguistic style than when the same individual communicates as a feminist. Further, the authors also show that individuals change their style based on the group that is psychologically relevant at the time of writing, even when demographics, personality and topic of communication are controlled for. Thus, individuals appear to switch between feminist and parent communication styles based on which of these two identities is psychologically relevant to them at a given point in time. In this way, we can see the direct link between the group that someone belongs to and the way in which they communicate when that group identity is psychologically relevant.

In the present research, we aim to explore whether group-based variation in communication style directly relates to a group’s collective self-understanding. More specifically, by examining the shared communication style of group members, we aim to understand the extent to which various groups differ in their underlying collective self-understanding. This, then, allows us to relate these similarities and differences to group types (Study 1) and values (Study 2), and to examine changes in collective self-understanding over time (Study 3).

## Study 1: Linguistic style reflects group type

Building on previous work from Koschate et al. (2021) and Cork et al. (2020), we are looking to understand whether similarities in group linguistic style map onto similarities in the collective self-understanding of the group and what the group stands for. In order to explore this idea, however, it is first necessary to have an understanding of which groups are perceived as having similar collective self-understandings. Previous research has used card-sorting tasks to assess the perceived similarities between different groups. In Deaux et al. (1995), the researchers found that individuals perceive there to be five different types of group. Using groups that are meaningful to individuals, such as ‘democrat, ‘aunt’ and ‘Asian American’, Deaux and colleagues asked participants to categorise 64 different social identities based on their perceived similarity. Five different types of social identity were observed: (a)vocational identities, political identities, ethnic and religious identities, relational identities and, finally, stigmatised identities. Vocational/avocational identities included groups such as teachers, psychologists, athletes and musicians; political identities included groups such as feminists, political independents, democrats and republicans; ethnic and religious identities included groups such as Jewish, Catholic, New Yorker and Asian American; relational identities included groups such as girlfriend, brother and divorcee; and finally, stigmatised identities included groups such as homeless people, fat people, gay people and people with AIDS.^1^

Based on the finding that individuals perceive five different types of group, here we explore whether this finding replicates using group members’ own linguistic style rather than outsiders’ (stereotypical) views of groups. That is, we examine whether a group’s collective self-understanding (in terms of the type of group they are) is reflected in a group linguistic style that shares similarities with other groups of the same type and differs from groups of a different type. In short, we hypothesise that identities from the same group type will use a more similar linguistic style than those from different group types. This hypothesis and the following methodology were preregistered at https://osf.io/jk6na/registrations.

### Method

#### Data collection

To assess our hypothesis, we chose three identities from each of the five group types outlined by Deaux et al. (1995). This allowed us to ensure that our choice of groups was both wide ranging as well as meaningful to individuals (Deaux, 1991). In line with the research of Koschate et al. (2021) and Cork et al. (2020), we used Reddit data to collect the linguistic style behaviour of our chosen identities. We chose to use Reddit data as the Reddit platform hosts forums for a diverse range of social groups. We assessed which of the groups used in Deaux and colleagues’ original 1995 research had suitable Reddit forums from which we could collect data. We chose three diverse identities from each of the five groups where the forum had a high number of users and an active Reddit community – these choices were preregistered at https://osf.io/jk6na/registrations.

Our aim when choosing the identities to include in this analysis was to ensure that we had a broad spread of different social groups. For the vocational category, we therefore chose one white collar vocation (r/sales), one self-employed vocation (r/entrepreneurs) and one more socially rather than economically focused vocation (r/teachers). These choices were limited by the Reddit forums that were available, for example r/lawyers and r/doctors were both private forums and thus we could not access the data within these subreddits. For the relational groups, we were limited again by the forums available. There were no active subreddits at the time dedicated to sibling relationships specifically, and so we chose mothers (r/breakingmom), fathers (r/daddit) and a general relationship forum (r/relationships). Here, we actively chose forums that appeared diverse and were not formed explicitly around relationship problems (e.g., r/justnofamily). For the ethnic and religious category, we chose identities that covered both ethnicities and religions. The r/asianamerican subreddit was one of the most active communities that we found dedicated to fostering community amongst individuals of a particular ethnicity. r/islam and r/Christianity were two of the largest active subreddits relating directly to religious groups, although other smaller communities were also available (e.g., r/Jewish). For the political groups, again many options were available. We attempted to find three forums that were politically diverse and had large active communities. We therefore chose one forum that relates directly to a political party (r/Conservative), one collective action group (r/feminist) and one group based on shared ideology rather than a specific political party (r/Libertarian). Finally, our choice of stigmatised identities to include in this analysis was constrained largely by the forums that exist with large active communities. For Deaux and colleagues original 1995 analysis, the authors had a few different types of stigmatised identities; those relating to sexuality (e.g., gay or lesbian), those with stigmatised traits (e.g., old, overweight or retired), those with potential substance use issues (e.g., alcohol or smoking), and those who are underprivileged (e.g., homeless, unemployed and welfare recipients). We aimed to mirror these choices but with groups that are currently relevant and active. Thus, we chose one identity from the LGBTQ+ community (r/asktransgender), one community confronting problematic substance use (r/stopdrinking) and one underprivileged community (r/homeless). All data used in this paper can also be found at https://osf.io/jk6na/. More detail about the subreddits from which we collected our data are outlined in Table 1.

**Table 1:** Information pertaining to the 15 subreddits used in analysis

Social group and type	Subreddit	Total users	Subreddit description
Entrepreneur (vocational)	r/Entrepreneur	~750 k	A community of individuals who seek to solve problems, network professionally, collaborate on projects and make the world a better place. Be professional, humble, and open to new ideas.
Salesperson (vocational)	r/sales	~135 k	Everything you need to know about sales, selling, business development, lead generation, prospecting, closing and more!
Teacher (vocational)	r/Teacher	~7.1 k	n/a
Asian American (ethnic/ religious)	r/asianamerican	~43 k	Anything related to Asian and Pacific Islander Americans, as well as other Asians who grew up outside of Asia. This includes news, discussions, pictures or videos. Whilst members of all races and nationalities are welcome, our purpose is to foster a sense of community among Asian Americans and their respective counterparts in the Asian diaspora. Topics do not necessarily need to be related to race as long as they contribute to the community
Muslim (ethnic/ religious)	r/islam	~132 k	r/islam is the place to discuss any topics related to Islam and Muslims
Christians (ethnic/ religious)	r/Christianity	~262 k	A subreddit to discuss Christianity and aspects of Christian life. All are welcome to participate
Libertarian (political)	r/Libertarian	~442 k	A place to discuss libertarianism, politics, related topics, and to share things that would be of interest to libertarians
Feminist (political)	r/Feminism	~195 k	Discuss and promote awareness of issues related equality for women
Conservative (political)	r/Conservative	~568 k	The place for Conservatives on Reddit
Mother (relational)	r/breakingmom	~61 k	Moms only. Just say what’s going on. No judgments, no nastiness
Father (relational)	r/daddit	~180 k	Subreddit for Dads. Single Dads, new Dads, Step-dads, tall Dads, short Dads and any other kind of dad
Partner/ friendships (relational)	r/relationships	~9 m	A community built around helping people and the goal of providing a platform for interpersonal relationship advice between redditors. We seek posts from users who have specific and personal relationship quandaries that other redditors can help them try to solve
Alcoholic (stigmatised)	r/stopdrinking	~261 k	This subreddit is a place to motivate each other to control or stop drinking. We welcome anyone who wishes to join in by asking for advice, sharing our experiences and stories or just encouraging someone who is trying to quit or cut down. Please post only when sober; you’re welcome to read in the meantime
Transgender (stigmatised)	r/asktransgender	~149 k	Transgender questions; transgender answers
Homeless (stigmatised)	r/homeless	~27.9 k	This is a discussion and advice group. Do not beg or soft-beg for cash, donations, etc.

After receiving ethical approval from the departmental ethics board and pre-registering the methodology and hypotheses, we collected 1 year’s worth of comments from the 15 subreddits listed above. Using Google BigQuery, we collected comments that had been posted to the aforementioned forums between January 2018 and January 2019. We collected the title, text, URL and anonymous user ID of all comments.

#### Data preparation

Following data collection, we quantified the linguistic data using Linguistic Inquiry and Word Count 2015 software (LIWC; Pennebaker et al., 2015). LIWC uses a bag-of-words language model, so that word order is ignored. It counts the number of words classified into particular linguistic categories, for example affective words, adverbs, future tense words (see Pennebaker et al., 2015, for further detail) and computes a percentage value for each document, reflecting the proportion of a particular feature in a document. Cork et al. (2020) and Koschate et al. (2021) have demonstrated that LIWC is a suitable software for understanding group normative linguistic styles.

For our analysis, we were interested in using only the LIWC categories that constitute linguistic style. We define style as the part-of-speech categories that are used widely across different contexts and domains regardless of topic (e.g., pronouns and articles; Schwartz et al., 2013). For this reason, we omitted all LIWC categories that refer to topical or content-based categories such as the ‘family’, ‘power’ and ‘risk’ categories. We also omitted the summary categories provided by the 2015 LIWC software that were an amalgamation of individual word categories such as ‘Clout’ and ‘Authenticity’ (Pennebaker et al., 2015). Resultantly, the textual data from each Reddit post was converted into a vector with 41 stylistic features (see Appendix 1).

Next, we excluded all comments made by self-identifying bots, all authors who have deleted their accounts or have had their accounts removed, all posts that have been deleted or removed, all posts that contain only a URL, and all comments with less than 50 words in line with common practice in computational psycholinguistic research using LIWC software (Cork et al., 2020). We also removed all URLs from the remaining comments. Table 2 indicates how many comments remained in our dataset after the data had been cleaned.

**Table 2:** Data remaining after excluding low-quality comments

Group type	Subreddit	Number of comments
Vocational	r/Entrepreneur	91,014
	r/Teachers	98,797
	r/sales	25,256
Ethnic/religious	r/asianamerican	17,519
	r/islam	54,495
	r/Christianity	356,604
Political	r/Conservative	134,767
	r/Libertarian	356,963
	r/Feminism	15,386
Relational	r/daddit	17,389
	r/breakingmom	87,323
	r/relationships	103,382
Stigmatised	r/asktransgender	207,527
	r/homeless	7,748
	r/stopdrinking	204,928

### Analysis and results

All analyses and results in this section was completed using Python 3.0. The code used for this analysis can be found at https://github.com/acork25/Identity-MDS.

#### Creating a dissimilarity matrix

In order to quantify the similarity between the linguistic styles of groups, we calculated pairwise models using machine learning algorithms. These pairwise models involved using a binary classification task to assess whether it is possible to train models that can learn to differentiate between the linguistic style of two social groups. For this research, we replicated the approach taken by Cork et al. (2020) and used Extremely Randomised Trees (“Extra Trees”) classifiers. Extra Trees classifiers choose the best feature-threshold combination for each split from a small randomly chosen set (Geurts et al., 2006). In this way, the Extra Trees model is less likely to overfit the training data through a more efficient method of reducing variance and bias within the dataset. Furthermore, due to the randomised procedure of splitting the data, Extra Trees are less computationally expensive (Geurts et al., 2006).

Imbalanced class sizes can adversely impact a classifier’s ability as merely choosing to classify every post as one of the majority class can still achieve an apparently high accuracy. In order to deal with the imbalanced class sizes of our dataset, we undertook random under-sampling. We selected the minimum number of comments for each pairwise class. The sample size for each pairwise comparison is listed in Table 3.

**Table 3:** Total sample size (N) included in each pairwise comparison (n = N/2)

	Entrepreneur	Sales	Teachers	Asian American	Islam	Christianity	Libertarian	Feminism	Conservative	BreakingMom	Daddit	Relationships	StopDrinking	Asktransgender	Homeless
Entrepreneur	182,028														
Sales	50,512	50,512													
Teachers	182,028	50,512	197,596												
AsianAmerican	35,036	35,036	35,036	35,036											
Islam	108,988	50,512	108,988	35,036	108,988										
Christianity	182,028	50,512	197,596	35,036	108,988	713,208									
Libertarian	182,028	50,512	197,596	35,036	108,988	713,208	713,924								
Feminism	30,772	30,772	30,772	30,772	30,772	30,772	30,772	30,772							
Conservative	182,028	50,512	197,596	35,036	108,988	269,532	269,532	30,772	269,532						
BreakingMom	174,644	50,512	174,644	35,036	108,988	174,644	174,644	30,772	174,644	174,644					
Daddit	34,776	34,776	34,776	34,776	34,776	34,776	34,776	30,772	34,776	34,776	34,776				
Relationships	182,028	50,512	197,596	35,036	108,988	206,764	206,764	30,772	206,764	174,644	34,776	206,764			
StopDrinking	182,028	50,512	197,596	35,036	108,988	409,856	409,856	30,772	269,532	174,644	34,776	206764	409,856		
AskTransgender	182,028	50,512	197,596	35,036	108,988	415,052	415,052	30,772	269,532	174,644	34,776	206764	409,856	415,052	
Homeless	15,496	15,496	15496	15,496	15,496	15,496	15,496	15,496	15,496	15,496	15,496	15,496	15,496	15,496	15,496

In total, 105 pairwise comparisons were completed using the Extra Trees classifiers. We included all 41 linguistic style features in the analysis (see Appendix 1). We divided the data for each pairwise class into a training set and a test set; we used 50% of the data to train the model and 50% of the data to test the model. We chose a 50:50 split as we used AUCs to reflect the extent of similarities/differences between the linguistic styles of groups, rather than just as an indication of classification accuracy. To ensure accurate estimates of similarities/differences, we opted for a larger test set than is common within machine learning research.

**Fig. 1:**
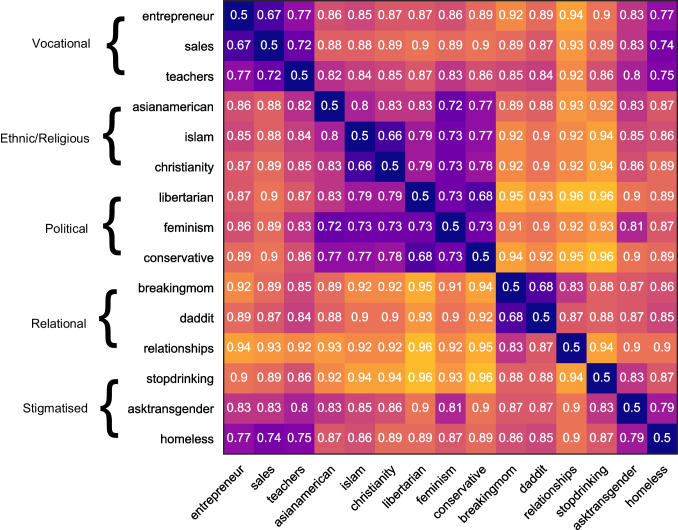
Shaded dissimilarity matrix consisting of AUC results (between-groups)

As mentioned, we used the resultant AUCs as a measure of dissimilarity between groups. Where two social groups had a more similar linguistic style, the AUC would be closer to 0.5 (= random guess) demonstrating that the classifier was less able to distinguish between the two identities. Conversely, for social groups with particularly distinct stylistic differences, the AUC of the Extra Trees classifier would be closer to 1.0 (= perfect separation). By using the AUC output of the Extra Trees classifier in all 105 pairwise comparisons, we constructed a dissimilarity matrix that illustrated how dissimilar each group was from each other (see Fig. 1).

#### Multidimensional scaling

**Fig. 2:**
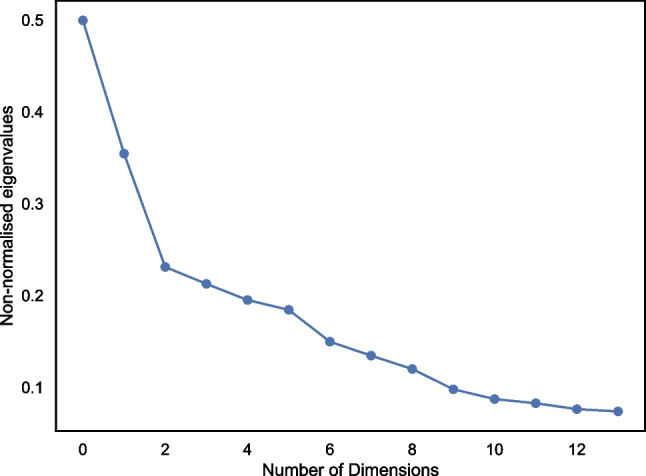
Eigenvalues scree (elbow) plot for MDS (multidimensional scaling) analysis

The next step was to run multidimensional scaling on the dissimilarity matrix to understand how the similarities between groups can be conceptualised in n-dimensional space. Specifying a Euclidean distance model, we computed and plotted the eigenvalues on a scree plot (Fig. 2), noting that two dimensions best fit the data (Kruskal & Wish, 1978).

**Fig. 3:**
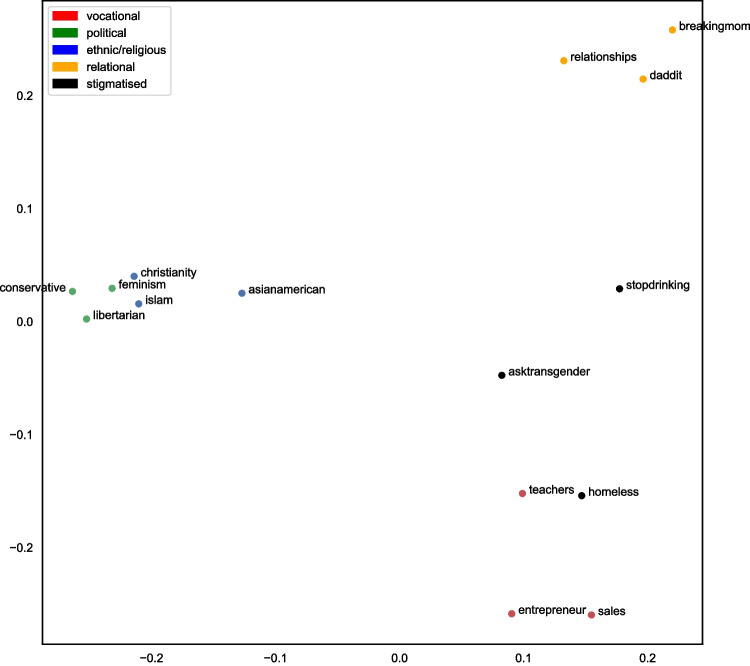
Multidimensional scaling plot illustrating the relations between the 15 different social identities colored by group type

We then plotted the two-dimensional multidimensional scaling (MDS) values on a scatterplot (Fig. 3). From Fig. 3, we can identify that our 15 identities appear to loosely cluster together into the five groups suggested by Deaux et al. (1995). That is, the identities that comprise each group type – vocational, relational, stigmatised, ethnic/religious and political – cluster relatively closely together on the two-dimensional plot. However, contrary to our predictions, the teacher identity and the homeless identity are notably close on the MDS plot. We explore potential reasons for this unanticipated result in the discussion below.

In Study 2, we explore the dimensions of the MDS plot in more detail; however, at this early stage it appears that the Y-axis represents a split on relational or empathetic characteristics, whereas the X-axis represents a split on a possible collective action focus. It is also possible that identities that fall towards the right-hand side of the plot are more advice-oriented, whereas the identities that fall towards the left of the plot are more opinion-oriented. In Study 2, we will assess how the values that these identities hold correspond to their placement on the axes.

### Cluster analysis

#### Hierarchical clustering

In our pre-registered methodology, we outlined a cluster analysis to examine whether similarities and differences in linguistic style differentiate sufficiently between types, in line with the methodology used by Deaux et al. (1995). Although the two-dimensional solution in our data already demonstrates some clustering, we performed hierarchical agglomerative cluster analysis on the dissimilarity matrix outlined in Fig. 1 in line with the pre-registration. Agglomerative clustering starts with each group identity as a singleton cluster and merges clusters successively based on their similarity. Similarity (or distance between clusters) can be calculated in multiple different ways (see Nielsen, 2016). However, in order to test the hypothesis that groups are more similar that share a group type than those that do not, we require a similarity measure that computes within-cluster variance. For this reason, we used Ward’s method, which aims to find the pair of clusters that have the lowest increase in within-cluster variance after merging (Nielsen, 2016). Ward’s method calculates the distance between two clusters by computing the increase in the sum of squares of two clusters when merged.

**Fig. 4:**
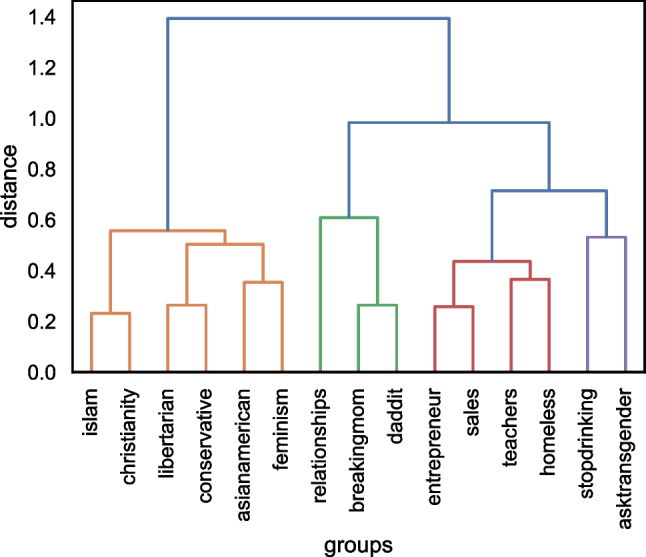
Dendogram of groups using hierarchical agglomerative cluster analysis (Ward’s method)

In Fig. 4, we note four clusters rather than the predicted five. Interestingly, we found that the homeless identity clusters with the vocational groups rather than with the stigmatised groups. Further, we also found that the political groups in our analysis cluster closely with the ethnic and religious groups. Nevertheless, we still observe an overlap between the group types found by Deaux et al. (1995) and the group types based on group members’ own behaviour.

#### K-means clustering

To further explore how the groups cluster, we conducted a k-means cluster analysis. As the agglomerative clustering is greedy procedure, meaning that clusters formed early in the clustering process cannot be separated in later steps, we used k-means clustering to refine the analysis of identity proximities.

**Fig. 5:**
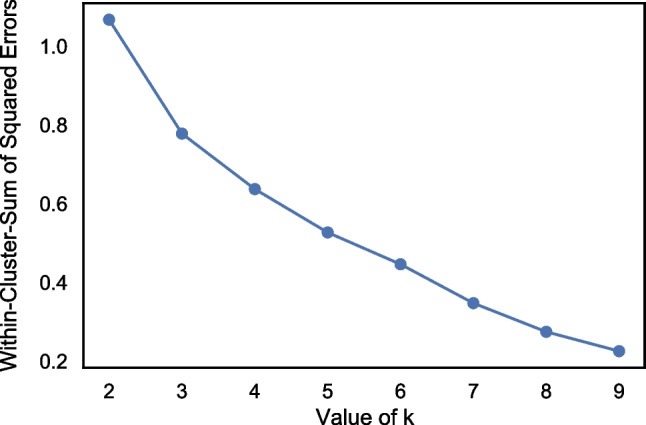
Elbow plot of within-cluster-sum of squared errors using k-means analysis

To determine the optimal value of k, we calculated the Within-Cluster-Sum of Squared Errors (WSS) for values of k from two to nine. After plotting the WSSs (Fig. 5), we found no clear threshold for the optimal number of clusters (there is no k where the WSS starts to level off). In line with the theory and as suggested by the agglomerative clustering, we chose k as 5 to assess whether the five group types suggested by Deaux et al. (1995) could be found within our data.

**Fig. 6:**
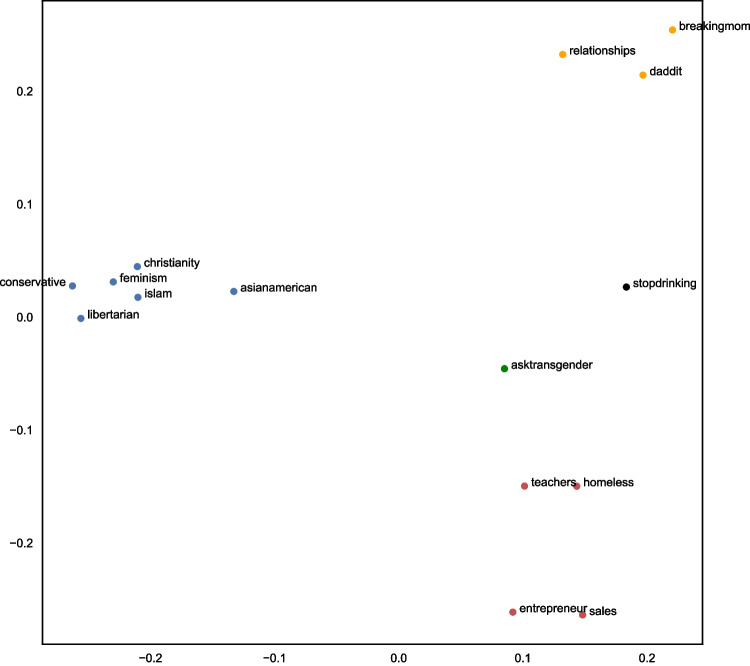
Multidimensional scaling plot with colour-coded clusters from k-means analysis where k = 5

Through using k-means cluster analysis (k = 5) on the dissimilarity matrix values in Fig. 1, we find that the five clusters do not map perfectly onto the five group types proposed by Deaux et al. (1995). Instead, we find that the first cluster consists of political and ethnic/religious groups, the second cluster is formed of relational groups, and the third cluster is formed of the vocational groups, including the homeless identity. Finally, the last two stigmatised groups form separate singleton clusters. Looking at the cluster-coloured MDS plot shown in Fig. 6, we can see that whilst there is notable overlap between the results of the agglomerative cluster analysis and the k-means analysis, the stigmatised groups do not cluster together at all. Possible reasons for this are explored in the *Discussion*.

### Discussion

The results from Study 1 tend to suggest that groups with similar collective self-understandings also have more similar linguistic styles. More specifically, we found that when using multidimensional scaling to visually map the linguistic similarities between group identities, we were somewhat able to identify the five types of group proposed in the perception-based research of Deaux et al. (1995); thus, the three social identities from each of our five group types were similar in their linguistic style and tended to be found closer together on the MDS plot. However, when we used agglomerative and k-means cluster analysis to further test the hypothesis that similarities within-group type would be greater than between-group type, the results were more mixed. In both the agglomerative and k-means clustering, we found that the homeless identity clustered with the vocational identities. It was also evident through looking at the MDS plot in Fig. 3 that the homeless identity was particularly close to the teacher identity. Further, in both the agglomerative and the k-means cluster analysis results, the political and religious/ethnic groups clustered together as one. Finally, the results of the k-means cluster analysis suggested that the linguistic style of stigmatised identities is more unique, with both the transgender group and problematic substance use group forming their own singleton clusters. Possible explanations for these findings are discussed below.

As can be seen on the dendogram in Fig. 3 and the k-means MDS clusters in Fig. 6, the political and ethnic/religious identities appear to form a single cluster. A likely reason for this clustering pertains to the meaning ascribed to particular ethnic and religious labels. To an outsider, or an individual who may not personally identify with the category label (e.g., the participants in Deaux and colleagues’ original 1995 study), ethnic and religious labels may be used as social labels to divide individuals into distinct groups on the basis of perceived physical and behavioural differences (McGarty, 1999). Thus, whilst an outsider may perceive similarities in groups such as ‘ethnicities’ or ‘religions’, this approach fails to comprehend what those group identities actually mean for the individuals and what the group stands for in practice. Herein lies the value of using naturally occurring behavioural data to study groups and group behaviour ‘in the wild’.

As has been argued elsewhere (Young, 2011), races, and by extensions ethnicities, are inherently political by the fact that they exist. Races are often defined in antithesis to the majority group; an individual may define someone as an Asian American in order to distinguish their ethnic identity from the superordinate American identity label. Conversely, the ‘White American’ is often perceived as the typical version of an American (Danbold & Huo, 2015; Devos & Banaji, 2005), and thus does not appear to require an explicit label. In this way then, the label ‘Asian American’ is used as a political tool to emphasise the boundary between Asian and White Americans. It therefore follows that the Asian American identity is political in nature, even if this is not recognised by laypeople such as those in Deaux et al.’s (1995) study. This argument is further supported by the lack of existence of a ‘White American’ or ‘European American’ Reddit forum. It is therefore no surprise that the Asian American group appeared closer to the overtly political identities than when only social judgments of outsiders were used to understand similarities between groups. This discrepancy between Deaux et al.’s (1995) results and our own points to the crucial value of using behaviour as a direct enactment of group identities in the real world, as opposed to relying merely on the stereotypical judgement of particular groups.

Interestingly, the closeness of the feminist identity and the Muslim identity in Fig. 2 also points to the value in using linguistic style to understand groups’ collective self-understandings. Despite the ideological differences between these groups, we can see that they both communicate in similar ways. It could be argued that the similarity between religious and political identities can be explained through understanding the fervently agentic nature of both identities (Deaux et al., 1995). More specifically, both religious and political identities are involved in collective activism with the intent of improving society; both identity types aim to create a lens through which to interpret human action, and as a result, a blueprint to improve upon society. In turn, our analysis as to how these identities are enacted goes beyond merely categorising individuals as similar (Deaux et al., 1995; McGarty, 1999; McGarty et al., 2002). By using behavioural analysis to understand who groups are and what they represent, we can directly capture how individuals construct their social realities within group-based environments (Reicher, 2004).

Relatedly, we noted that the homeless identity mapped very closely to the teacher identity and was clustered with the vocational identities in both the agglomerative and the k-means clustering. One explanation for this finding pertains to the purpose of the r/homeless subreddit. For individuals posting in this subreddit, it is possible that their communication is focused more around how to earn a living and survive whilst homeless, rather than on the stigmatised nature of their identities. In this way, the action-based focus around how to make money to survive is more likely to resemble the linguistic style of vocational groups rather than the other stigmatised groups (r/stopdrinking and r/asktransgender).

In addition to this, we can also see from results of the k-means analysis that the stigmatised identities have less of a distinctive linguistic style than the other group types. The k-means cluster analysis demonstrated that the transgender and problematic alcohol use groups formed their own singleton clusters rather than being clustered together. This is likely due to the greater variation in what it means to be part of a stigmatised group. Through looking at the AUCs outlined in Fig. 1 this explanation makes sense. The stigmatised identities have much lower AUCs when compared to all identities (including other stigmatised identities). This suggests that they are not particularly similar to any of the groups included in this analysis; the most similar group to the transgender group is the homeless group (AUC = .79), the most similar group to the problematic alcohol use group is the transgender group (AUC = .83), and the most similar group for the homeless group is sales (AUC = .74). Thus, whilst for two out of three stigmatised groups their closest group is another stigmatised group, they are still not that similar, with AUCs over .79. For future research, it would be interesting to include more stigmatised groups to understand how they fit within this framework. As noted earlier, when we chose the different groups to include in our analysis, we attempted to maximise diversity. We noted that in Deaux et al.’s original analysis (1995), the authors had three different types of stigmatised group – underprivileged, LGBTQ+, and problematic substance use. It is therefore possible that these different types of stigmatised groups each have their own linguistic profile. For future research, this could be a worthwhile avenue to explore.

However, one key strength of the research pertains to the diversity of the groups chosen to be included in this analysis. As outlined earlier in the *Methods* section, we chose three identities from each group that embodied diverse representations of the group type. For example, we included a diverse array of political groups (based on a political party, a collective action movement and an ideology), a diverse array of vocational groups (white-collar, self-employed and socially focused) and a diverse array of stigmatised groups (LGBTQ+, economically underprivileged, and confronting problematic substance use). It is therefore quite interesting that despite the broad range of topics that is likely being discussed within these 15 communities, we still observe some clustering in line with our predictions. This strength also points to the value of using linguistic style to assess groups’ collective self-understandings instead of linguistic content.

At this point, it bears noting that in this research the context is an online public forum. It is likely that this specific context will have an impact on how individuals choose to communicate and the purpose of their communication. Having said this, whilst this specific context may indeed play a key role in impacting the purpose of communication and the style of group communication, we suggest that this is a ‘feature’ and not a ‘bug’ of this methodological approach. We do not aim to deny the role of context and purpose, but instead argue that the purpose of communication is intrinsically tied up with the purpose, values and collective self-understanding of the group. That is, if two groups communicate in similar ways because they share a common purpose (i.e., supporting others or trying to mobilise the group for action), we note that this represents the collective self-understanding of the group and is thus central to understanding the similarity between different groups online.

In the next study, we look to more closely map the way a group communicates with the explicit values that the group holds. Whilst at present we know that the clusters represent group types in line with those perceived by participants in Deaux et al. (1995), our second study uses the linguistic content of groups’ discussions to demonstrate the link between linguistic style and group-based values.

## Study 2

In order to validate the idea that group linguistic style maps to similarities in a group’s collective self-understanding, this study ascertains whether the position of each group on the MDS plot (Fig. 2) corresponds directly with the values that each group holds. Conceptually, therefore, we are aiming to explain each group’s position in multidimensional space by using the value-based content of each group’s interactions. Study 2 uses the same data as Study 1 to further understand the results of Study 1; it is therefore a secondary analysis.

Previously, research has suggested that individual-level values can be reliably identified using automated text analysis (Boyd et al., 2015), and thus we aim to extend this finding to assess values at the group-level. Through comparing naturally occurring written communication, self-reported values and value-laden behaviours, Boyd et al. (2015) find strong support for language-based value-behaviour links. In fact, they note that when values are operationalised through employing linguistic analysis on naturally occurring social media posts, they are better able to predict an individual’s future behaviour than when using self-reported scales. Building on this research, Ponizovskiy et al. (2020) developed a simple and easy-to-use value lexicon that is based on Schwartz’s theory of basic values (Schwartz, 1992). Schwartz’s theory of basic values (1992) suggests that there are ten values that ‘form a quasi-circumplex structure based on the inherent conflict or compatibility between their motivational goals’ (Schwartz & Boehnke, 2004, p.203). The values suggested by Schwartz are: benevolence, universalism, security, conformity, tradition, self-direction, stimulation, hedonism, power, and achievement. Ponizovskiy et al. (2020) find that their vocabulary-based dictionary has high reliability and a pattern of correlations between values that shows synthesis with the circumplex structure of values proposed by Schwartz (1992). Whilst this specific dictionary-based tool has not yet been used to assess social group values, previous research has demonstrated the benefit of using Schwartz values more generally to understand and conceptualise the values of particular social groups (e.g., Saroglou et al., 2004). In light of this, we suggest that the dictionary-based tool may be well suited to understanding the values of groups online.

We therefore use the value-based dictionary developed by Ponizovskiy et al. (2020) to assess the values of our 15 groups. We then assess whether the values of each group can be used to predict their position on the MDS plot. This analysis will help validate the idea that the way in which a group communicates corresponds with the values that are part of a group’s collective self-understanding.

### Method

**Fig. 7:**
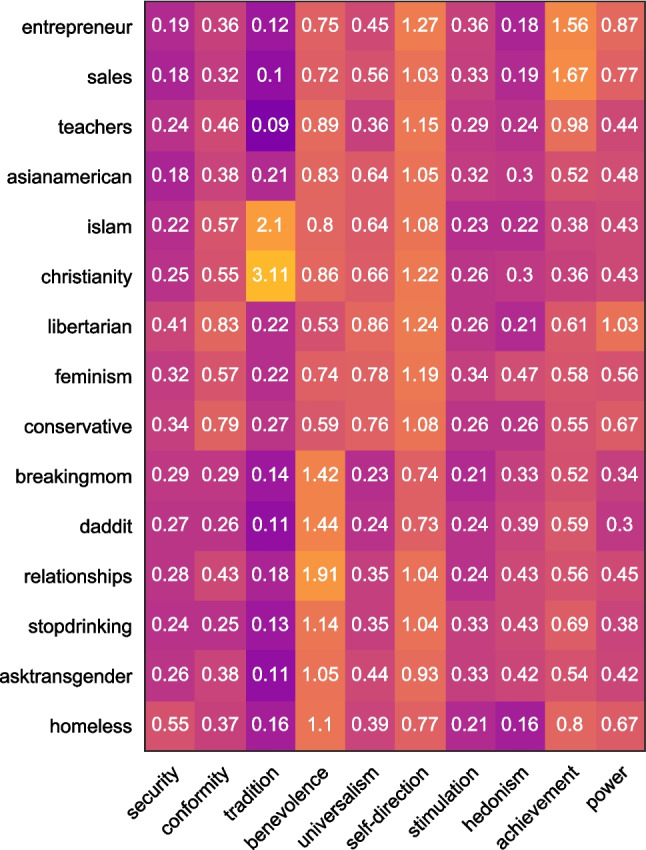
Mean percentage of words from each group relating to ten Schwartz values

In order to assess the values held by each of our 15 groups, we ran the open-access dictionary provided by Ponizovskiy et al. (2020) on the dataset outlined in Study 1 (*N* = 1,779,098). For each of the ten Schwartz values, we calculated a score for each post that referred to the total percentage of words relating to that value. Using the mean value score for each group, we calculated ten value scores for each of the 15 identities. These are displayed in the matrix of Fig. 7.

In the next step, we aimed to understand whether the values that the groups discussed (i.e., the value content) correlated with their location on the MDS plot (which is based on their linguistic style). Following the analysis by Deaux et al. (1995), we ran Ordinary Least Squares (OLS) regressions separately for each of the ten Schwartz values, whereby the coordinates from the MDS plot were used to predict the groups’ values. Hence, our sample consists of *n* = 15 groups. The outcome variable of each regression is one of the ten Schwarz values, and the predictor variables are the x- and y-coordinates of the MDS plot from Study 1. The beta-coefficients resulting from our ten OLS regressions are listed in Table 4.

**Table 4:** Multiple regression of value scores on multidimensional scaling coordinates for 15 groups

Values	Unstandardised regression weights						*R*^*2*^	*p*
	Dimension 1 (x-axis)			Dimension 2 (y-axis)				
	B	*SE*	95% CI	B	*SE*	95% CI		
Achievement	0.84*	0.30	0.19, 1.50	-1.88***	0.36	-2.65, -1.11	.75	<.001
Benevolence	1.30***	0.23	0.80, 1.79	1.48***	0.27	0.89, 2.07	.84	<.001
Conformity	-0.82***	0.14	-1.11, -0.53	-0.01	0.16	-0.36, 0.34	.76	<.001
Hedonism	0.07	0.12	-0.20, 0.33	0.42*	0.14	0.11, 0.74	.42	.037
Power	-0.36	0.24	-0.88, 0.15	-0.78*	0.28	-1.40 -0.17	.46	.026
Self-direction	-0.57**	0.18	-0.97, -0.17	-0.44	0.22	-0.92, -0.03	.53	.010
Security	-0.05	0.15	-0.36, 0.27	0.04	0.17	-0.34, 0.42	.01	.924
Stimulation	-0.02	0.07	-0.16, 0.13	-0.17	0.08	-0.34, -0.00	.29	.127
Tradition	-2.27	1.17	-4.82, 0.28	0.56	1.39	-2.47, 3.58	.25	.183
Universalism	-0.99***	0.09	-1.18, -0.79	-0.26*	0.11	-0.50, -0.03	.91	<.001

*p<.05 **p<.01. ***p<.001

*SE* standard error, *CI* confidence interval

**Fig. 8:**
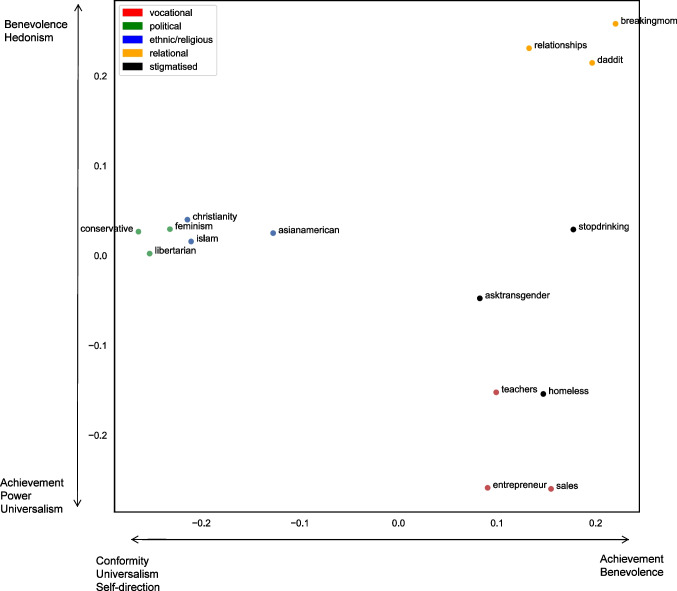
Multidimensional scaling plot with Schwartz values corresponding to axes

From Table 4, we can see that groups that value achievement tend to be found towards the bottom right corner of the MDS plot, reflecting the positive correlation with the x-axis (dimension 1) and the negative correlation with the y-axis (dimension 2). As can be seen in Fig. 8, this is where our vocational groups are situated. Conversely, groups higher in benevolence tend to be found in the top right corner of the plot – as both the x- and y-axis show a positive correlation with benevolence. This is where our relational groups are situated. Meanwhile, groups higher in conformity, self-direction and universalism tend to be found towards the left side of the plot (corresponding to a negative correlation with the x-axis), where our political and religious groups lie. Additionally, the y-axis values are positively associated with hedonism, and negatively associated with power. This suggests that our relational identities may be more hedonistic, whereas our vocational identities may be more concerned with power.

### Discussion

The results of this analysis demonstrate that a group’s content-based values are related to the position on the MDS plot, that is, the group’s self-understanding. This provides evidence to support the idea that individuals are expressing the collective self-understanding of their groups through the way in which they communicate online. By comparing each group’s linguistic style with another group’s linguistic style, we can therefore gain an idea of the type of group it is, as well as the collective self-understanding and underlying values of the group that drive their communication style. However, it is worth noting however that the regression analyses used here were only conducted on 15 datapoints – one for each of the 15 groups used in the analysis. Due to this low sample size, it is important to interpret these results with caution. We suggest that future research would benefit from repeating this analysis with more diverse groups to understand whether this initial finding replicates.

The results indicate that groups which value achievement tend to be found near where our vocational groups are situated. This shows synthesis with the self-reported findings of Deaux et al. (1995, Study 2) who found that individuals were more likely to perceive vocational groups as ‘achieved’, as opposed to ‘ascribed’. In Deaux et al.’ (1995) Study 2, the researchers also looked to explain whether certain attributes could be used to predict the MDS coordinates of the 64 groups included in their initial analysis. Deaux and colleagues used indicators such as whether a group was perceived to be individual or collective, ascribed or achieved, and desirable or undesirable. In total, they had 15 different traits that were used to understand the position of the groups.

Similarly, the results of the value regression analysis demonstrates that groups high in benevolence tend to be found towards the top right corner of the plot towards our relational groups. This makes intuitive sense given that relational groups are more care-focused as compared to other types of groups. However, it is also interesting to note that variations in benevolence may also help to understand within-cluster variations. For example, we found that the ‘teacher’ group appears to be higher up the y-axis than the other vocational groups. Here, we can see that whilst teachers are clustered with other vocational groups, their position within this cluster may be explained through understanding their values, in this case the centrality of benevolence to the teaching profession (Neufeld & Hargreaves, 1999). This argument may also help to understand the position of the religious groups on the plot, as compared to the political groups; whilst both types of group may be associated with collective action – at least, in an online forum context – it could be argued that benevolence plays a more fundamental role in understanding religion and religious groups than it does in political groups more generally (Lee et al., 2013).

The other strong correlations between the Schwartz values and the position of the groups on the MDS plot pertain to conformity and universalism. Specifically, we find that groups high in conformity and universalism tend to be located towards the left side of the plot. Again, this aligns with research of Deaux et al. (1995) who found that individuals tended to rate political, ethnic and religious groups as being more collective. Deaux et al. (1995) suggest that these identities connote some form of common group membership in a way that the other types of identity (stigmatised, vocational and relational) do not.

In sum then, the relationship between the values that are reflected in the content of group discussion online and their position on their collective self-understanding, as represented in the linguistic style-based MDS plot, appears to show good face validity. This points to the value of using linguistic style to understand groups in real-world contexts. Further, by using style rather than content directly, we can access the inherent values of the group even when the topic of conversation may be non-normative or masked (Koschate et al., 2021).

The final contribution of this behavioural methodology is to demonstrate its applicability to studying groups online. Next, we show the value of this approach not just for understanding the similarity between groups as a cross-sectional snapshot, but also for understanding the development and evolution of a group’s collective self-understanding over time.

## Study 3

In our final study, we aim to understand whether our method can be used to study the development of groups over time. In order to understand whether our methodological framework could be used to observe subtle shifts over time, we chose to focus on the evolution of the transgender identity over the past decade. As has been argued by the American news website Vox, ‘few marginalised communities have experienced such a dramatic whiplash of fortunes over the course of the 2010s as trans people’ (Burns, 2019). Or, as stated in the British newspaper The Spectator, ‘the 2010s were the decade of trans’ (Emmons, 2019). These mainstream news articles point to the major developments, both legally and culturally, that trans rights movements and transgender individuals have achieved over the past decade (2010–2020). We therefore believe that this makes the transgender identity a suitable case-study for understanding whether we can use our linguistic style/MDS approach to track the evolution of groups over time.

Whilst the transgender rights movement has been in existence since the 1980s/90s (Stryker et al., 2008), the legalisation of same-sex marriages in the Western world during the early 21st century led LGBTQ+ movements to shift from focussing on gay rights, to focussing on transgender rights (Green, 2016; Taylor et al., 2018). For example, in 2016, Obama’s Social Security Administration passed a law making it easier for transgender individuals to amend their passports and legal records (White House Press Office, 2016). In the UK, the Equality Act of 2010 declared gender reassignment as a protected characteristic, thus making it illegal to discriminate against transgender individuals in professional environments. Further, the World Health Organization also withdrew transgender health issues from the ICD-11, proclaiming that transgender issues are no longer considered as mental or behavioural disorders (World Health Organization, 2019). Whilst not all rulings over the past decade have been in favour of transgender rights – for example, the Trump administration’s ban on transgender individuals serving in the military – it is clear that transgender rights and issues have gone from the political fringe to the political mainstream (Taylor et al., 2018). It is therefore possible that the transgender identity is becoming more political over time.

Similarly, the 2010s decade observed a greater visibility of trans individuals in mainstream media (Green, 2016). In June 2014, Time Magazine dedicated its front page to the ‘Transgender Tipping Point’, with the accompanying article discussing the growing awareness of trans rights (Steinmetz, 2014). Additionally, Olympic medallist Caitlyn Jenner appeared on the cover of Vanity Fair, after coming out as trans in 2015 (Bissinger, 2015). Further, whilst it is clear that transgender individuals have been more visible in the media since 2010, research by Austin and Goodman (2017) has suggested that the increased media attention and political centrality of transgender individuals has led to a positive shift in the social and cultural attitudes towards trans people. Furthermore, statistical research has demonstrated a steady increase in the proportion of individuals identifying as transgender and non-binary in both Europe and the USA (see Nolan et al., 2019, for a review). This increase in the number of individuals identifying as transgender may point to the idea that transgender identities are slowly becoming less stigmatised. Based on this historical and social context then, it appears that the transgender identity is slowly becoming more politicised whilst simultaneously becoming less stigmatised within mainstream society.

Despite this positive social shift, it is important to acknowledge that transgender individuals still report high levels of stigmatisation both during and following their transition (Bry et al., 2018; Miller & Grollman, 2015; Verbeek et al., 2020). As a result of this stigmatisation, the prevalence of depression and anxiety in trans individuals exceeds that of the general population (Witcomb et al., 2018). Whilst there is a plethora of qualitative research that has focused on the stigmatising experience of transgender individuals within the past decade, it is difficult to assess whether there have been collective-level changes in the way transgender individuals perceive their own group. That is, we do not currently know whether transgender groups’ collective self-understanding has been impacted by changes in political and legal rights, and perceptions in some parts of mainstream society.

In this study, therefore, we explore this idea using our novel linguistic style methodology. Based on the increasing political success of the trans rights movement (Taylor et al., 2018) coupled with the increasing social acceptance of transgender individuals by the general population (Austin & Goodman, 2017), we hypothesise that we will be able to observe a shift in the linguistic style behaviour of transgender groups, for example in the transgender group r/asktransgender on Reddit. Specifically, we suggest that the position of the Reddit transgender group on the MDS plot will shift from being closer to the stigmatised identities at the beginning of 2012 towards the collective action identities by 2019. That is, we hypothesise that using only linguistic style features, we will be able to observe the gradual politicisation and de-stigmatisation of the transgender identity between the years 2012 and 2019.

### Method

#### Data

In order to understand the evolution of the transgender group identity over time, we collected Reddit data dating back to 2011 using Google BigQuery. In line with our previous analysis, we collected comments from the subreddit r/asktransgender, a forum consisting of transgender individuals discussing their personal experiences and identity. We cleaned these data to remove any bots, authors that had been removed, comments that had been deleted, or comments with less than 50 words. Following data cleaning, the total number of comments included in the analysis is shown in Table 5.

**Table 5:** Data collected from the r/asktransgender subreddit over 9 years

Year	No. of Reddit posts
2011	15,620
2012	31,823
2013	51,596
2014	84,294
2015	149,808
2016	183,092
2017	201,570
2018	207,589
2019^a^	177,139

^a^The 2019 data only include posts up to October 2019 as this was the most recent data available on Google BigQuery at the point of collation

### Analysis and results

#### Multidimensional scaling analysis

After running the data through LIWC software to quantify the linguistic style of each post (see Appendix 1), we repeated the MDS analysis outlined in Study 1 nine times, each time with a different year’s worth of data from the transgender forum. In this way, all the other groups were held constant so that we could be sure that any movement of the position of the transgender group on the MDS plot could be attributed to changes in the transgender group specifically.

To repeat the MDS analysis, we first created a dissimilarity matrix between the yearly transgender sample and the other 14 identities. We took the pairwise minimum training posts and testing posts between each year of transgender data and each of the 14 identities (in line with Study 1). We computed the dissimilarity between the transgender group and the other 14 groups using the Extra Trees classifier as before. Once again, we entered all 41 stylistic variables into the analysis (see Appendix 1).

**Fig. 9:**
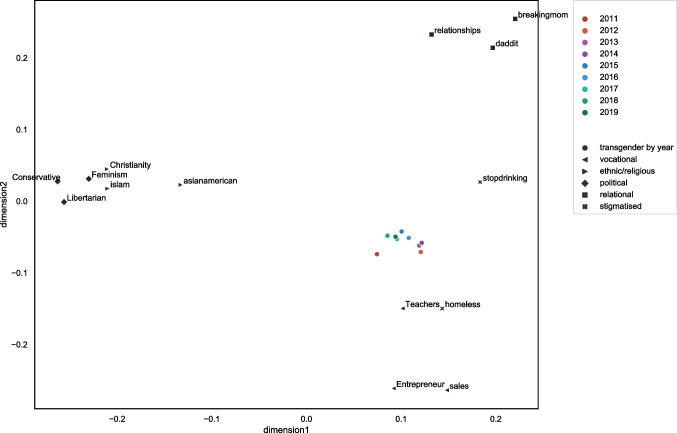
Multidimensional scaling plot illustrating the evolution of the transgender group over time

To complete the MDS analysis, we included only 1 year’s worth of data at a time. The coordinates for each year’s worth of data are plotted in Fig. 9. As outlined in the legend, the colour of the transgender points refers to the date of the data. The redder the colour, the earlier the data.

As we can see from Fig. 9, there is evidence to suggest that the transgender group is ‘moving’ away from the more stigmatised groups, and towards the collective action groups on the left side of the plot. To further understand the statistical significance of the relationship between the position on the MDS plot and the year of posting, we ran a correlational analysis.

#### Correlational analysis

In order to understand the relationship between the placement of the transgender identity on the MDS plot and the year of posting, we ran a Spearman’s correlational analysis. We looked at the correlation between the year of posting (2011–2019) and the placement of the transgender identity on the MDS plot (the coordinates of each years’ worth of transgender data). Whist our correlational analysis only had nine datapoints, it is important to remember that each of these nine points has been calculated through, first, conducting 105 pairwise comparisons, and then using MDS analysis on the resulting dissimilarity matrices to generate the coordinates for each year’s worth of data.

**Fig. 10:**
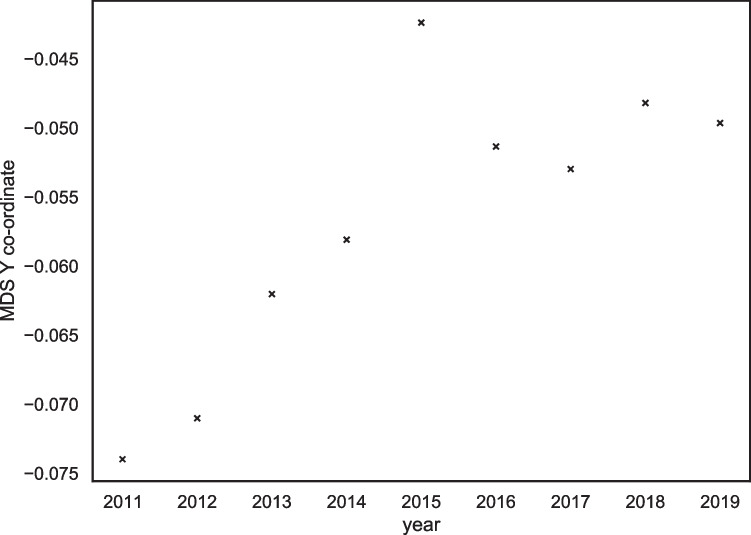
Scatterplot demonstrating the positive relationship between the multidimensional scaling Y coordinate and year of transgender data

**Fig. 11:**
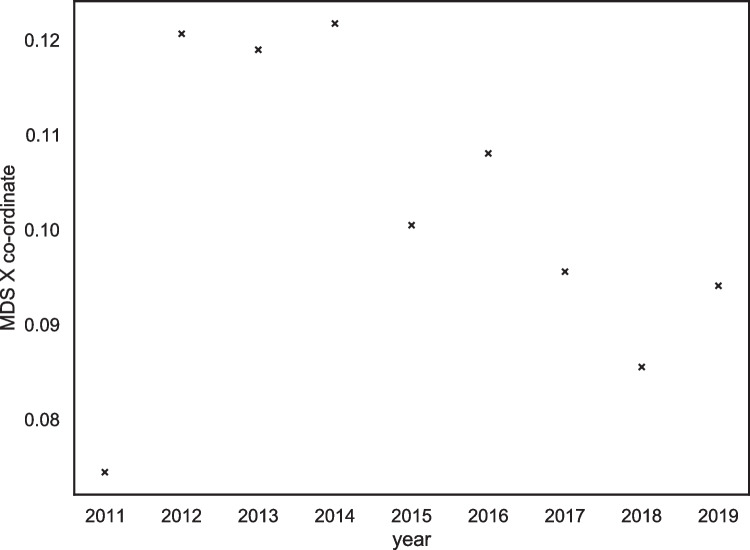
Scatterplot demonstrating the positive relationship between the multidimensional scaling X coordinate and year of transgender data

At first, we noted a significant correlation between the year of posting and the Y coordinates, *r*(7) = .80, *p* = .007, (see Fig. 10); however, no significant correlation between the year of posting and the X coordinates, *r*(7) = -.32, *p =* .53 (see Fig. 11). This finding suggested that the transgender group was ‘moving’ towards the top the MDS plot where the relational groups are, although was not moving towards the left side of the plot where the collective action groups sit.

After visually inspecting the scatterplot of the X coordinates and the year of posting however, we identified that 2011 appeared to be an anomalous year (see Fig. 11). This may be because the year 2011 was when the forum was first established and thus the community was not developed at this point. We repeated the analysis omitting the 2011 data. We found that when the 2011 data were excluded from the analysis, a strong and significant negative correlation was observed between the year of posting and the X coordinate, *r*(6) = -.88, *p =* =.002. This finding aligns with the visual inspection of the MDS plot in Fig. 9. We also noted a significant positive correlation between the year of posting and the Y coordinate, *r*(6) = .71, *p* =.04. Thus, after removing the anomalous 2011 data from the analysis, the correlational analysis supported the hypothesis that the transgender identity was moving towards the collective action groups on the MDS plot that are situated further to the left on the X-axis and slightly more towards the top on the Y-axis compared with the stigmatised cluster.

### Discussion

The significant correlations between the MDS coordinates and the year of the r/asktransgender Reddit data provides compelling evidence to suggest that this approach can be used to track the development of groups and their collective self-understanding over time. Here, we have used r/transgender as a proof-of-concept case to demonstrate the value of this approach. We have shown that changes in the linguistic style of r/transgender appear to correspond with the gradual politicisation and de-stigmatisation as reported in both mainstream media headlines and the results of qualitative, self-reported research (Taylor et al., 2018). This result thus provides support for the hypothesisthat suggested that it would be possible to use changes in linguistic style to observe the changes in the collective self-understanding of groups. Further, this finding points to the value of using linguistic style and the MDS approach outlined in this paper to understand online group development without needing to manually code linguistic data. This approach could therefore be used in triangulation with more fine-grained qualitative analyses.

We note that the correlation between the MDS axes and the year of posting is strong, although the movement of the group is slow. This is in line with research from cultural evolution theory which suggests that groups tend to adopt new norms and identities slowly through processes of active negotiation and passive transmission (Mesoudi, 2016). Whilst some behavioural and cultural variants are adopted and integrated with groups, others are dropped quickly, or may never be fully adopted. It is therefore not unexpected that the transgender group does not move significantly faster across the MDS plot but is instead slow yet consistent.

The results of this study thus demonstrate an exciting application for using MDS approaches and linguistic style to study group behaviour online. We believe that this approach has multiple applications for both psychologists and other social scientists, as well as implications and uses in government organisations. For example, for social psychologists and political scientists, it may be possible to understand leadership processes through ascertaining which leaders are able to change the overall collective self-understanding of the group. Whilst this notion has been well studied using self-reported data (Reicher & Hopkins, 2003), there has been little research that has attempted to understand leadership processes using naturally occurring behavioural data. Moreover, by using naturally occurring online data, these research aims can be achieved in relatively inexpensive ways that are not impacted by the usual barriers to longitudinal research such as high rates of attrition. We can also access historical data which can be used to understand the collective self-understanding and values of present-day social groups.

## General discussion

Through the three studies presented in this methodological paper, we have demonstrated the value of using linguistic style analysis to measure and track groups’ collective self-understanding over time. In the first study, we showed that groups’ communication styles correspond to perceived group similarities. We noted that groups that were perceived as more similar to each other also demonstrated more similar linguistic style behaviours. In the second study, we highlighted the link between groups’ linguistic style behaviours and the values that they held. Specifically, we showed that the values that groups hold are directly correlated to their position on our MDS plot. Finally, we applied this approach to understand how groups may develop over time. In synthesis with the results of qualitative, self-reported research (Taylor et al., 2018), we found that the transgender group was slowly becoming less stigmatised and more politicised over time and that this could be observed using our MDS plot using naturally occurring online data.

In sum, the results from these three studies provide compelling evidence that suggests that linguistic style can be used to understand the collective self-understanding of social groups. In this research, we think of the collective self-understanding as comprising the abstract norms, values and purpose of the group rather than the specific opinions or content of the groups’ beliefs. For this reason, groups with similar abstract values yet different opinions (e.g., conservatives and feminists) still present as more similar than they are to other types of groups (Deaux et al., 1995). In turn, this methodological approach provides an automated way of understanding who a group is by comparing them to other types of groups. This can allow us to study groups who are less accessible due to their inherent nature (e.g., criminal groups).

Through using naturally occurring online data to study group behaviour, we are able to transcend many of the limitations associated with self-report methodologies. Specifically, we are able to directly examine how groups enact their values and their collective self-understanding in practice. We are therefore not concerned with the way that groups present themselves to researchers, or the values that they purport having. Instead, we focus on how individuals adopt group labels in practice and thus enact their values in real-world contexts. As we have seen from research on the values of individuals (Boyd et al., 2015), the values that individuals purport having are less predictive of their value-based behaviour than the values that they communicate through their language. Whilst not directly tested within this paper, this finding from Boyd et al. (2015) points to the importance of studying what groups stand for through an analysis of their language, rather than relying on them to accurately self-report their values.

Through using naturally occurring linguistic data, this approach can be used to study and compare the collective self-understandings of a vast array of groups ‘in the wild’ and how they change over time. By understanding the group type that particular groups belong to, we can gain a greater understanding of their motivations and collective identity. For example, the initial Incel website (involuntary celibate) started off as a relational support group for individuals struggling with their relationships. However, over time this group has become a politicised anti-feminist group with misogyny at its centre (Jones, 2020). Some Incels have even taken their misogyny offline through engaging in violent attacks (e.g., Plymouth, UK, 2021). Using an automated methodology like the one outlined in this paper, it may be possible to identify the politicisation of groups. This could allow both political scientists and governmental bodies to monitor this politicisation before it escalates into radicalisation. Whilst the method outlined in this paper has not yet modelled the behaviour of radical groups, we believe that this could be a very interesting avenue for future research.

At present, emerging political movements are often studied in a retrospective manner. It is only when a political movement gains traction that time and resources are invested in understanding their collective self-understanding of the new movement and what the group stands for. Often, this research is qualitative, and thus it can take several years from the inception of the group before researchers are able to publish their findings. However, we suggest that through using an automated approach that relies on naturally occurring data, we can shorten this process and direct attention earlier to groups of interest. Whilst our approach is not suited for identifying specific attitudes or opinions that groups hold, it nonetheless underscores the general values of the group and thus complements more fine-grained qualitative analysis.

Whilst there are various applications for understanding the politicisation of groups, this approach can also be used to study harder to access groups such as criminal organisations. We know there to be a plethora of data about criminal groups available on both darknet forums as well as surface web forums (e.g., Gab; Moriarty, 2017). However, at present, given the number of potential criminal group forums that exist online, law enforcement may struggle to identify exactly which groups or forums are worth allocating resources to. We suggest that the approach outlined in this paper may allow law enforcement officials to have a better understanding of the values and collective self-understanding of different groups, which can then enable resources to be allocated more efficiently. Whilst there may be some criminal groups that are more akin to relational support groups (e.g., certain substance use forums), others may be identified as more business-oriented groups thus serving as a greater criminal threat (see Cork, 2021). Again then, we stress the ability of this approach to act as an automated, low-cost early warning system.

Outside of applications within law and politics, this approach offers exciting new avenues for social scientists to study group behaviour more generally in naturally occurring contexts. For example, through developing this methodology, it may be possible to understand how within-group variance impacts social dynamics such as emotional contagion or information dissemination (e.g., Felfernig et al., 2018). For example, using only self-reported data, Gelfand et al. (2011) have demonstrated that tight and loose group norms have important implications for tolerance towards group deviant behaviour (Gelfand et al., 2011), in turn impacting outcomes such as responses to the Covid-19 pandemic (Gelfand et al., 2021). We therefore suggest that the methodological approach proposed in this paper may allow us to gain greater insight into the normative behaviour of real-world groups online and may thus prove important for studying the social dynamics associated with online groups. Through gaining greater insight into the collective self-understandings of online groups, this could aid in the mitigation of certain harmful group behaviours, such as the spreading and belief of conspiracy theories.

Whilst we have focused on naturally occurring forum data within this paper, we suggest that this approach could also be used on other types of linguistic data. For example, it may be possible to map how political speeches have developed and evolved over time, and to understand how this relates to changes in the collective self-understanding of certain political groups. This could enable social scientists to understand how political groups develop in relation to leader’s speeches; does the leader direct the change of the group’s collective self-understanding, or does the group move first and the leader follows (Hollander, 2012)? By using real world data rather than self-reported data, we can access group-based phenomena that individual group members may not be consciously aware of and thus may be unable to self-report.

It is clear that there are many future applications and developments for this research approach and methodology. However, we also need to acknowledge some of the limitations associated with this work. Firstly, it is challenging to distinguish between platform-specific norms and group-specific norms. In the studies above, we used only Reddit data in order to control for the impact of platform effects on group normative behaviour. However, it is necessary to be aware of the potential role of the platform in determining socially normative behaviour (e.g., Hanusch, 2017). In turn, we must be cautious not to overgeneralise these results to groups that are not included in the analysis (e.g., non-Reddit transgender groups). Another limitation pertains to the language used to perform the analysis. At present, this research has only been conducted using the English language and thus it is not clear whether the same patterns would emerge should this analysis be replicated in other languages. Whilst we have no reason to suspect that the findings would not hold in other languages, the impact of language on the results is not currently clear.

In conclusion, this paper presents an automated methodological approach for assessing groups' collective self-understandings using textual data. We suggest that this approach has many potential applications for both social scientists and governmental agencies alike.

## Data Availability

Data are available at: https://www.osf.io/jk6na/.

## Appendix 1: LIWC linguistic style categories

Outlined in Appendix Table 6 are the LIWC stylistic categories used in this research. These categories are part-of-speech categories that are used widely regardless of topic.

**Table 6:** 41 LIWC stylistic variables used initial analysis

Pronouns	Function words	Other grammar	Psychological	Time	Perception/relativity	Structural/ informal
First person singular	Articles	Adjectives	Affect	Past focus	See	Words per sentence
First person plural	Prepositions	Verbs	Insight	Future focus	Hear	6 letter words
Second person	Auxiliary verbs	Interrogatives	Causation	Present focus	Feel	Swear
Third person singular	Adverbs	Numbers	Discrepancy		Motion	Netspeak
Third person plural	Conjunctions	Quantifiers	Tentative		Space	Assent
Impersonal pronouns	Negations	Comparisons	Certainty		Time	Non-fluencies
			Differentiation			Fillers

## Footnotes

1 Group names taken directly from Deaux et al. (1995)
